# A Review on Adducin from Functional to Pathological Mechanisms: Future Direction in Cancer

**DOI:** 10.1155/2018/3465929

**Published:** 2018-05-16

**Authors:** Karrie Mei-Yee Kiang, Gilberto Ka-Kit Leung

**Affiliations:** Department of Surgery, Li Ka Shing Faculty of Medicine, The University of Hong Kong, Queen Mary Hospital, Pok Fu Lam, Hong Kong

## Abstract

Adducin (ADD) is a family of membrane skeleton proteins including ADD1, ADD2, and ADD3 that are encoded by distinct genes on different chromosomes. Adducin is primarily responsible for the assembly of spectrin-actin network that provides physical support to the plasma membrane and mediates signal transduction in various cellular physiological processes upon regulation by protein kinase C-dependent and calcium/calmodulin-dependent pathways. Abnormal phosphorylation, genetic variations, and alternative splicing of adducin may contribute to alterations in cellular functions involved in pathogenic processes. These alterations are associated with a wide range of diseases including cancer. This paper begins with a discussion on how adducin partakes in the structural formation of membrane skeleton, its regulation, and related functional characteristics, followed by a review on the pathogenesis of hypertension, biliary atresia, and cancer with respect to increased disease susceptibility mediated by adducin polymorphism and/or dysregulation. Given the functional diversity of adducin in different cellular compartments, we aim to provide a knowledge base whereby its pathophysiological roles can be better understood. More importantly, we aim to provide novel insights that may be of significance in turning the adducin model to clinical application.

## 1. Introduction

Although adducin functions primarily as a membrane cytoskeletal protein, it is also an essential molecule in maintaining a wide range of physiological functions. Aberrant alterations of adducin resulting from genetic, transcriptional, epigenetic, and posttranslational modifications could be fatal. While its pathogenic role in hypertension has been known for decades, its role in other diseases including biliary atresia and cancer was recognized only in recent years. This review summarizes the characteristics of adducin based on its structure and function, its dysregulation, and involvement in different disease pathogenesis. Recent findings on adducin in cancer will also be discussed including its controversial roles as an oncoprotein and tumor suppressor. A better understanding of these particular properties of adducin may provide valuable insights into direct future studies in the identification of novel biomarkers and possibly new therapeutic tools.

## 2. Adducin in Membrane Cytoskeleton

**Figure 1:**
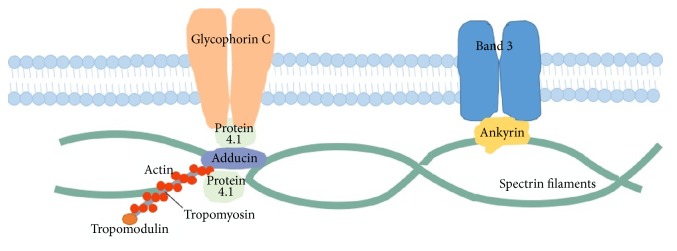
*The structural proteins in the spectrin-based membrane skeleton at the cytoplasmic surface of plasma membrane*. Spectrin is a rod-shaped protein that binds with actin filaments at each end. Spectrin and actin associate with accessory proteins (ankyrin and protein 4.1) at the junctional complex and connect with tropomyosin and tropomodulin to form a polygonal structure in the membrane cytoskeleton. Glycophorin C and Band 3 are the integral membrane proteins involved in the spectrin-based skeleton conferring the membrane with strength and deformability.

Membrane skeleton was first studied in mammalian red blood cells. The latter are nonnucleated cells with a supported lipid bilayer and the ability to withstand high shear force within the circulatory system [1]. This lipid-based membrane structure was later extracted and studied [2]. On electron microscopy, the membrane skeleton was found to consist of long spectrin filaments that are interconnected by actin-containing junctions, thus forming a scaffold of polygonal network [3, 4] (Figure 1).

This spectrin-actin lattice forms connections and attaches to plasma membrane through two protein-protein linkages. The first linkage is mediated by ankyrin, which binds to the cytoplasmic domain of transmembrane protein Band 3 and *β*-spectrin [5–7]. The second linkage is through protein 4.1 that binds transmembrane protein glycophorin C on its cytoplasmic domain and binds to the N-terminus of *β*-spectrin [8, 9], thus linking the spectrin skeleton to the membrane. A similar interaction that links the spectrin skeleton with plasma membrane has recently been identified between adducin and glucose transporter-1 [10]. Protein 4.1 also exerts its function at spectrin-actin junctions [11–13], where it interacts with other cytoskeleton proteins including tropomodulin, tropomyosin, protein 4.9, and adducin and regulates the formation of spectrin-actin network [14, 15]. Besides providing physical support to the cell membranes, the spectrin-based membrane skeleton is also involved in the regulation of membrane trafficking [16], formation of signaling complex and cell adhesion complex [17], movement of membrane proteins [18–20], and cell motility [21].

Back in the early 1970s, spectrin was found to interact with actin alone at a very low affinity [22–24], raising the question of how spectrin and actin were able to form the cytoskeletal network mentioned above. It was later found that the assembly between spectrin-actin was facilitated by two proteins: protein 4.1 [11, 12] and adducin [25–27]. Unlike protein 4.1, adducin is preferentially associated with spectrin-actin complex as compared to spectrin or actin alone and is a crucial assembly factor that recruits and promotes the association of spectrin to actin [28, 29]. Named by its ability in ‘adducing' the formation of spectrin-actin complex, adducin acts as an assembly factor for the formation of spectrin-actin membrane skeleton. As it self-associates, it forms an actin-binding domain located at the extended tails to bundle with actin filaments at the sides [27], while the flexible tail allows adducin to bind with large molecules including spectrin and actin filaments simultaneously [30]. Such assembly of the spectrin-actin lattice is facilitated by adducin in an ordered pathway reaction. Beginning with the binding of spectrin to actin, adducin then associates itself with the spectrin-actin complex, recruits additional spectrin to the assembly lattice, and bundles and caps the fast-growing barbed end of actin filaments to prevent the addition or loss of actin subunits [27–29, 31–33]. Other accessory proteins mentioned earlier are also required for the assembly of spectrin-actin network in membrane skeleton.

## 3. Adducin Structure, Subunits, and Localization

Adducin is encoded by three different yet closely related genes [34, 35], referred to as *α*-, *β*-, and *γ*-adducin or ADD1, ADD2, and ADD3, respectively, mapping to three different chromosome locations. It is expressed as heterotetramers in either *α*/*β* or *α*/*γ* heterodimeric combinations. Gardner and Bennett were the first group to isolate and identify the membrane-associated protein adducin from red blood cell cytoskeleton based on its calmodulin binding activity [25]. Adducin is associated exclusively with plasma membranes and is detected in a wide range of tissues [29, 36, 37]. Other than red blood cells that contains 50 pmol per mg of membrane protein, brain tissue has the most abundant adducin (12 pmol per mg membrane protein) among all other tissues, suggestive of certain specialized functions of adducin in nerve cells [29]. While *α*- and *γ*-adducins are ubiquitously expressed and *α*/*γ* heterodimers are found in platelets and most other nonerythroid cells [35, 38], *β*-adducin is tissue-specific and restricted to the brain and hematopoietic tissues as *α*/*β* heterodimers [34, 35, 39]. It has been reported that the physical and functional properties of red blood cell adducin and brain adducin are indeed closely related [29].

Polypeptide mapping of adducin has revealed the structural and functional properties of different domains [30]. Adducin subunits are related in sequence and have similar domain structures, containing an N-terminal globular head domain, a neck domain, and a C-terminal protease-sensitive tail domain that is homologous to myristoylated alanine-rich C-kinase substrate (MARCKS) protein [30]. Li et al. described the importance of subunit oligomerization necessary for its activities and observed that adducin does not exist as monomers but forms dimers or tetramers in its neck and probably head domains [40]. Such head-head and even tail-tail contact was shown to be essential in maintaining the oligomeric conformation of equilibrium [30, 34]. Among the three subunits, the 22-residue MARCKS-related tail domain is highly conserved and contains calmodulin binding sequence as well as abundant protein kinase A/protein kinase C (PKA/PKC) phosphorylation sites [30, 34]. Phosphorylation sites for PKA and Rho-kinase have also been identified at around the neck domain of adducin subunits; however, similar phosphorylation site is lacking in the head domain, rendering it unable to bind calmodulin or interact with spectrin and actin [30]. The structural characteristics of the MARCKS-related tail domain are essential for its interactions with other proteins, suggesting a role in mediating spectrin-actin assembly through the fundamental regulation of adducin functions.

Cellular localization of adducin is compatible with its roles in various cellular functions. For example, adducin is localized at spectrin-actin junctions in mature red blood cells and is expressed during early erythropoiesis [41, 42]. It is also concentrated at sites of cell-cell contact in epithelial tissues, but restricted to the lateral cell borders of intestinal epithelial cells. It is expressed in cultured cells such as keratinocytes and Madin-Darby Canine Kidney (MDCK) cells and serves as a constituent in synaptic structures. Moreover, adducin is highly expressed at dendritic spines, platelets, and axon growth cones in cultured neurons [29, 36, 43, 44]. Recent evidence shows that adducin could be subjected to phosphorylation and also to be redistributed and translocated to nucleus possibly in both phosphorylated and unphosphorylated form [45, 46]. However, redistribution of adducin appears to be calcium-calmodulin-dependent that is in turn affected by PKA, PKC, and Rho-kinase phosphorylation, with properties similar to those of the MARCKS protein family. Redistribution or translocation of adducin offers a diversity of functional roles apart from maintaining cytoskeletal stability [47]. More details about the functional roles of adducin are described in the following sections.

## 4. Adducin Regulations and Functions

The heterogeneous functions and activities of adducin appear to be dependent on its C-terminal MARCKS-related tail domain, which has similar sequence with high homology with the MARCKS protein family. The MARCKS family of proteins are well-known as the predominant substrate for PKC [48, 49]; they are localized in the plasma membrane and able to bind and cross-link actin filaments. Phosphorylation by PKC or binding to calcium-calmodulin, however, can inhibit its actin-binding activity, causing it to redistribute to the cytoplasm. Besides, MARCKS proteins may also associate with plasma membrane through electrostatic interactions between its basic residues and the acidic phospholipids of the membrane such as phosphatidylserine. This interaction was also shown to be affected upon phosphorylation whereby the electrostatic binding of MARCKS proteins to phospholipids would be reduced and thus dissociate from the membrane [50, 51].

Adducin are functionally associated with MARCKS proteins. The MARCKS-related tail domain of adducin is required for its activity in forming the spectrin-actin network of membrane skeleton [40]. It was shown that its spectrin-recruiting activity would be abolished when the ionic composition was disturbed, suggesting that the tail domain mediates the direct contact with spectrin-actin through electrostatic interactions [40]. Like the MARCKS proteins, adducin is a substrate of various protein-kinases such as PKC, PKA, and Rho-kinase and is regulated in a phosphorylation-dependent and calcium-calmodulin-dependent manner [52]. Ser-726 in *α*-adducin and Ser-713 in *β*-adducin are the major phosphorylation sites common to both PKC and PKA [53]. Phosphorylation of adducin by PKC and PKA generally inhibits the actin capping, actin binding, spectrin-recruiting activity, and calmodulin binding [53]. Conversely, phosphorylation by Rho-kinase enhances adducin-actin interactions and spectrin recruitment [54, 55]. Changes in intracellular calcium could alter PKC activation and secondarily influence adducin phosphorylation. Since the dominant calmodulin binding site was also found in the MARCKS-related tail domain of adducin, changes in calcium level may result in the inhibition of calmodulin binding by PKA/PKC phosphorylation. However, the binding of calmodulin could in turn inhibit phosphorylation events by PKA/PKC, thus forming a reciprocal regulation of adducin [53]. Major functional consequences of adducin phosphorylation are listed in Table 1.

While the activity of adducin in spectrin-actin assembly might be modulated upon phosphorylation, the phosphorylated form of adducin has been reported with diverse functions at other localizations. Phospho-adducin redistributed to the cytoplasm is known to play important roles in morphological regulation during platelet activation [38, 56], localizing cell-cell contacts in epithelial cells [36, 57], the formation of membrane such as lamellipodia [58], stabilizing endothelial adhesion junctions [59], establishing cell-cell junctions [60, 61], and modulating cell motility [55, 58]. Translocation of phospho-adducin to the nucleus has been studied. Chen et al. identified the nuclear localization signal (NLS) in the tail region immediately adjacent to Ser-716 and the nuclear export signal (NES) in the neck region of *α*-adducin, indicating an inherent mechanism for adducin to shuttle between cytoplasm and nucleus. A recent literature has reported the different nuclear export abilities between three adducin isoforms. While *α*-adducin was suggested to have the potential role in regulating transcription, it was found to interact with RNA polymerase II and zinc-finger protein 331 [62]. Interestingly, reduced cell-cell adhesion together with impaired cell proliferation characterized by mitotic defects was observed when *α*-adducin was depleted [46, 63]. During mitosis, *α*-adducin appeared to be crucial for proper mitotic spindle assembly. It could be phosphorylated by cyclin-dependent kinase 1 (CDK1) at Ser-12 and Ser-355 and subsequently bind to myosin-X that would enable it to associate with mitotic spindles [63]. Another study also demonstrated that Ser-713 and 726 of *β*-adducin translocated to the nucleus may associate with heterochromatin and centrioles to stabilize their structures during mitosis [64]. The evidence suggests that adducin may play different regulatory roles in cell proliferation machinery in accordance with its subcellular localization. However, due to the fact that some of the studies on adducin did not distinguish between the phosphorylated and unphosphorylated form, it could be possible that those works focused on total adducin that could also have similar functions.

Alterations of cell morphology have been shown to convey cell shape signals to control cell growth [65, 66]. Disruptions of a cell's cytoskeleton can inhibit cell-cycle progression in G_1_/S transition [66–68], and an intact cytoskeleton is essential for endothelial cells or fibroblasts to passage through late G_1_ and subsequent S phase entry [69–72]. It is likely that the cell shape signals conveyed by adducin may exert considerable impact on the biochemical signaling machinery in controlling cell proliferation.

## 5. Adducin and Related Disorders

Given its functional versatility and involvement in different biological processes, it is not surprising that adducin is implicated in disease pathogenesis. Adducin participates in signaling transduction machinery upon regulation at different levels, and there is mounting evidence to suggest that dysregulations of adducin could lead to various diseases including cerebrovascular diseases, gastrointestinal disorder in infants, cardiovascular diseases, and cancer.

### 5.1. Hypertension

Essential hypertension is characterized by a chronic elevation in blood pressure without a specific medical or biological cause [74, 75]. Patients with hypertension have common defects in renal tubular function including enhanced constitutive renal sodium reabsorption and impairment in renal sodium excretion [75]. Early experimental studies on hypertension have shown that the red blood cells in Milan hypertensive strain of rats (MHS) were smaller than those in the normotensive control (MNS) and with a faster rate of ion transport [76]. To detect the subtle differences between the membrane skeletons of MHS and MNS, membrane skeleton from MHS was injected into MNS and vice versa. MHS rats immunized with membrane skeleton of MNS red blood cells produced anti-adducin antibodies, suggesting a genetic or structural difference in adducin that may contribute to altered ion transport and possibly the subsequent development of hypertension [76]. The identification of adducin also led to a long series of studies aimed at delineating its role in the initial pathophysiological triggering mechanism of hypertension. Experimental data suggested that abnormalities in red blood cell membrane skeleton were genetically determined within stem cells [77] and that hypertension is closely related to the genetic variations of membrane skeleton proteins.

#### 5.1.1. Adducin Polymorphism in Hypertension

Despite over 20 years of research and the use of large-scale genome-wide association studies (GWAS) in the identification of candidate genes in essential hypertension, only modest association was found. This is in part limited by genetic heterogeneity and epitasis in this multifactorial disease. Whole genome scanning has provided us with a clue that single nucleotide polymorphism (SNP) may be involved in linking gene polymorphisms to blood pressure phenotype [78–81]. Adducin polymorphism in the *α*-subunit (*Gly*460*Trp*) (ref SNP cluster ID, rs4961) is one of the few candidate gene polymorphisms affecting blood pressure both in rats and in humans. Clinical impact of adducin polymorphisms in hypertension and related disorders has been extensively studied in different experimental settings and across different populations [82–113]. Results from experimental, clinical, and epidemiological studies indicate that genetic variants of *α*-adducin, and* Gly*460*Trp* in particular, contribute significantly to the pathogenetic mechanisms of hypertension.

Sequence analysis on full-length cDNA adducin in MHS and MNS rats revealed point mutations in MHS adducin subunits *α*-*Phe*316*Tyr*, *β*-*Gln*529*Arg*, and *γ*-*Gln*572*Lys* [114, 115]. Chromosomal region of the* Add1* locus in MHS transferred to MNS strain could induce a high blood pressure phenotype and vice versa [116]. Adducin polymorphisms were also seen in human genome at ADD1-*Gly*460*Trp* (rs4961), ADD1-*Ser*586*Cys* (rs4963), ADD2-*Cys*1967*Thr* (rs4984), and ADD3-IVS11+386*Ala*>*Gly* (rs3731566) [117]. Among these polymorphisms, the ADD1-*Gly*460*Trp* gene variant was identified as a candidate gene for hypertension. In human case-control association studies, ADD1 gene with the mutant allele (460*Trp*) showed an increased risk of hypertension and a reduced sodium content in red blood cells with a faster rate of ion transport than those with the wild-type ADD1 460*GlyGly* homozygote allele [117, 118]. In line with clinical studies, renal tubular reabsorption was increased in untreated hypertensive patients who carry the mutated 460*Trp *allele compared to those with 460*GlyGly* homozygote [119]. Many epidemiological studies have since been conducted to evaluate the association of* Gly*460*Trp* variant with hypertension across different populations [85, 86, 88, 90, 95, 105, 108, 109, 112, 118, 120–123]; the magnitude of its impact was found to be variable when environmental-biological-genetic factors were also considered. High dietary salt intake is also a risk factor for hypertension, and blood pressure responses to dietary salt could be influenced by adducin genetic variations. For example, individuals with an 460*Trp* allele were genetically predisposed to salt-sensitive hypertension [117, 124].

*In vitro* and* in vivo* studies based on ADD1 mutation have unraveled the primary molecular mechanism that underlies the transition from normotension to hypertension in the context of sodium handling. Both the mutated* Add1* gene (*Phe*316*Tyr*) in rat and the mutated ADD1 gene (*Gly*460*Trp*) in human affect protein functions that increase Na^+^/K^+^ pump (also known as Na^+^/K^+^-ATPase) cellular expression and activity in renal tubular cells as well as Na^+^/K^+^ pump-dependent signal transduction [125–127]. Furthermore, the mutant gene also impaired Na^+^/K^+^ pump endocytosis that would enhance renal salt reabsorption and ultimately lead to high blood pressure [119, 120].

The interactive relationship between adducin polymorphism and ouabain, an endogenous hormone that modulates Na^+^/K^+^ pump activity, appears to play a role in regulating sodium homeostasis and hypertension. Plasma levels of ouabain increase in parallel with the copy number of the mutant 460*Trp* allele [128], through which the renal Na^+^/K^+^ pump activity is modulated by this mutant phenotype. Further efforts are required to unmask the interactions between 460*Trp* allele and other environmental-biological-genetic factors such as salt intake, age, and genetic patterns (e.g., physiological interaction with adducin subunit genotypes ADD2 and ADD3, ACE polymorphism [129], WNK1 polymorphism, and NEDD4L polymorphism) [130] in the pathogenesis of hypertensive [131–136]. A more detailed review on adducin polymorphism in hypertension has been described by Citterio et al. [137].

### 5.2. Adducin Dysregulation in Other Diseases

The significance of the ADD1 460*Trp *allele in other conditions has also been described, including cerebrovascular disease such as hemorrhagic stroke [90, 103], cardiovascular disease such as atherosclerosis [138] and myocardiac infarction [139], and renal diseases [99, 101, 113]. Additionally, ADD1* Ser*617*Cys* polymorphism (rs4963) was reported to be associated with hypertension in the Asian population [140]. Intriguingly, recent research has identified this variant in association with an increased susceptibility to several human malignancies including noncardia gastric cancer and colorectal cancer [141, 142]. It has been reported that ADD2 (rs4983) variant in combination with sodium-calcium exchanger 1 (NCX1) variants (rs11893826 and rs434082) could possibly interact in regulating systemic inflammation and influence the risk of system lupus erythematosus [143]. Mutation in ADD3 is associated with inherited cerebral palsy due to impaired neuromotor activity [144]. Through GWAS across different populations, SNPs (rs17095355, rs10509906, and rs7099604) identified at chromosome 10q24 (ADD3 and XPNPEP1 loci) were also shown to increase the susceptibility for biliary atresia (BA) [145–149]. Genetic factors may underpin pathogenesis of BA, which is a devastating disease of the liver and bile ducts characterized by fibroobliteration and obstruction of extrahepatic biliary system in the first few weeks of life [150]. Several studies have been conducted to elucidate pathogenic pathways of BA. A study using zebrafish model has demonstrated that the loss of* add3a,* but not* xpnpep1*, could lead to impaired biliary function and intrahepatic defects possibly through suppressing hedgehog pathways [151]. In addition, upregulated expression of ADD3 is also reported to contribute in liver fibrosis in BA and associated with downregulation of miR-145 which targets ADD3 [152].

How adducin dysregulation can lead to such a diverse range of disorders is incompletely understood. One potential mechanism involves a linkage between the epigenetic modulation of adducin expression and clinical phenotypes. In essential hypertension, a case-control study found that the extent of ADD1 promoter methylation was inversely associated with the risk of hypertension in a gender-dependent manner. Lower level of methylation led to a higher expression of ADD1 protein regardless of genotype, leading to increased Na^+^/K^+^ pump activity and salt reabsorption [153]. By the same token, targeted deletion of individual adducin subunits could lead to various abnormalities.* Add1*,* Add2*, and* Add3* null mice have been generated as mammalian disease models, each of them are presented with specific phenotypes [154].  Table 2 listed the characteristics associated with the animals and implied the possible functions of these proteins* in vivo*.

For instance, *α*-adducin is the limiting subunit in oligomer formation and *α*-adducin deficiency would abolish the protein expression of *β*- and *γ*-adducin in red blood cells. This may result in significant growth retardation with a hereditary spherocytosis red blood cell phenotype and hydrocephalus in *α*-adducin knockout mice [155]. Surprisingly, however, in* in vivo* studies involving *β*- and *γ*-adducin null mice, red blood cells were able to retain intact membrane skeleton and normal hematological parameters despite a reduced *α*-adducin protein level [39, 156]. Consistent findings were seen in *β*- and *γ*-adducin knockout models, indicating that *α*-adducin is required to partner with *β* or *γ* subunit for the stability and functioning of red blood cells and that the loss of either *β* or *γ* subunit would compensate the loss of the other subunit to maintain the normal red blood cell phenotype. It is noteworthy that mice with *γ*-adducin depletion are phenotypically normal with only a slight reduction in *α*-adducin protein level, but *β*-adducin knockout mice are presented with various functional disorders. This phenomenon has raised the intriguing possibility that *α*-adducin may function as homodimer or homotetramer and compensate for the loss of *γ*-adducin in different tissues, except in tissues such as brain and hematopoietic cells where *β*-adducin is uniquely expressed, and *α*-adducin may not be able to function and compensate for the loss of *β*-adducin.

In the brain, *α*-adducin is highly expressed in dendritic spines and growth cones of neurons as constituents of synaptic structures and necessary for maintaining periodic structure and diameter in axons [44, 157]. Adducin knockout mice models have already demonstrated the functional roles of *α*-adducin in CSF homeostasis [155], *β*-adducin in synaptic plasticity in hippocampus modulating motor coordination and learning/memory process, and *γ*-adducin in promoting outgrowth of neurite as well as budding of secretory protein vesicles from the Golgi network [158, 159]. Since adducin forms actin branching and stabilizes synapses with its actin capping activity, its dysregulation may alter neural network and memory maintenance [160]. Alterations in phosphorylated adducin expression have been reported in patients with amyotrophic lateral sclerosis (ALS), implying that adducin may be involved in the regulation of pre- and postsynaptic stability at neuromuscular junction [161].

In the kidney, *γ*-adducin plays important role in modulating renal salt reabsorption through its interaction with the thiazide-sensitive NaCl cotransporter, thereby influencing blood pressure homeostasis [162]. *γ*-Adducin has recently been revealed to partake in renal and cerebral circulations through the regulation of vascular myogenic response [163]. Lastly, *γ*-adducin might direct angiogenesis [164], suggesting that it might play important roles in cancer progression. Taken together, these findings indicate the role of adducin in regulating and maintaining the dynamics of cell membranes.

### 5.3. Cancer

There are only a limited number of studies that worked on the functional properties of adducin in cancer, and recent works have largely focused on its role in drug resistance, tumorigenesis, and tumor metastasis. Here, we summarize the key findings and examine some of the controversies.

#### 5.3.1. Adducin in Drug Resistance

Györffy et al. performed microarray analysis across 30 different cancer cell lines treated with 11 different anticancer drugs and identified ADD3 as one of the candidate genes for multidrug resistance [165]. ADD3 was also reported to be associated with chemoresistance in osteosarcoma [166]. Another* in vitro* study described an increase in ADD3 expression in temozolomide resistant glioblastoma cells and that its expression is colocalized with CD133 in glioma stem-like cells, suggestive of a positive correlation between ADD3 and cancer stem cell phenotype as well as chemoresistance [167].

#### 5.3.2. Adducin in Tumorigenesis and Metastasis

Adducin also takes part in oncogenic signal transduction pathways in various cancers. It was found that ZNF322A, previously identified as an oncoprotein, could promote tumor cell growth and metastasis in lung cancer via ADD1 and CCND1; knockdown of ADD1 would suppress lung cancer cell migration and invasion [168]. Moreover, the growth suppressive effect of forced miR-145 overexpression in glioma cells is likely to be mediated through the suppression of Sox9 and ADD3 proteins [169]. Another study reported that *α*-adducin-Ser724 and *γ*-adducin-Ser662 were both downstream signal transduction molecules of c-MET pathway implicated in small-cell lung cancer invasion and metastasis [170].

Considering that numerous signaling pathways which involve PKC are being activated during cancer progression, an increased PKC-mediated phospho-adducin level is likely to occur in cancer cells. Indeed, alterations in adducin expression, localization, and phosphorylation states have been observed upon malignant transformation. In renal cell carcinoma, adducin was found to be associated with tumor progression, characterized by a reduction in total adducin level but increased *γ*-adducin-Ser660 phosphorylation. The change in phosphorylation level correlated with changes in cellular distribution from the apical-basal membranes to the lateral membrane in proximal tubular cells [45, 171]. High levels of PKC*δ* in mammary tumor cells could similarly increase its metastatic potential [172]. The ability of PKC*δ* to promote tumor cell motility may be the result of increased adducin phosphorylation, which could enhance cell migration and in turn tumor metastasis [58].

Notably, Shen et al. suggested the notion that the phosphorylation-related variants of adducin are involved in tumorigenesis. For instance, the substitution of a serine residue by a cysteine in* Ser*586*Cys* in ADD1 could prevent its phosphorylation, influence its activity in proliferation, and increase susceptibility to colorectal cancer [141]. SNPs that alter amino acids may also influence posttranslational modifications including phosphorylation and therefore protein function. Taken together, these research findings are suggestive of adducin's roles in oncogenic pathways and cancer progression. As loss of adducin phosphorylation was seen in bone marrow and tumor samples of cancer patients treated with cyclin-dependent kinase inhibitors [173], chemotherapeutics targeting adducin may potentially be exploited as novel treatment strategy for these patients.

That said, microarray gene expression profiling studies have found that ADD3 mRNA expression was in fact significantly downregulated during glioma progression when compared to its less malignant or nonneoplastic counterparts [174, 175]. Another microarray analysis also found a reduced ADD3 expression in relation to increased migratory activity in glioma cells [176]. These microarray data on glioma specimens are in sharp contradictions with what have been described in the foregoing sections which include mainly cell-based studies. One possible explanation of this apparent discrepancy could be due to differences in microenvironment. We surmise that adducin could preferentially acts as an oncoprotein in a two-dimensional cell-based setting, where its expression would correlate negatively with tumor progression. By contrast, adducin may act as a tumor suppressor in a three-dimensional microenvironment found in whole-tumor specimens. Newly published data from Lechuga et al. has demonstrated the function of adducin in negatively regulating cancer cell motility and invasion [177]. Without having any effects on cell proliferation, stable knockdown of either ADD1 and ADD3 increased migration of non-small-cell lung cancer cells (NSCLC), and overexpression of ADD1 reduced its migration and invasion activities. It was suggested that the negative effect of ADD1 overexpression in cell motility could be mediated by the enhanced adhesion to extracellular matrix (ECM), as well as remodeling of the actin-cytoskeleton [177]. Indicating that the interaction of adducin with the ECM or the microenvironment could be an important factor when considering its functional roles.

#### 5.3.3. Alternative Splicing of Adducin in Cancer

Another possible explanation for the above discrepancies could be due to the differential expression of ADD3 transcript isoforms generated from alternative splicing. Two different splicing isoforms, ADD3a and ADD3b, have been identified early in 1999. Citterio et al. was the first to report the genomic organization of adducin in which ADD3a transcript contained exons 12, 13, and 14, while exon 13 was absent in ADD3b [137]. In a recent study using exon array, transcript variants of ADD3 have been further validated and found to be differentially spliced between non-small-cell lung cancer (NSCLC) and normal lung tissue. Of the 16 exons in ADD3 gene, the cassette exon, exon 15 (ENSE00000986819), is preferentially expressed in NSCLC but not in normal lung tissue [178]. In line with this, ADD3 containing the cassette exon was found to be highly expressed in the highly metastatic 4T1 murine breast tumor when compared to the nonmetastatic 168FARN tumor, in which the cassette exon was missing [179]. It was concluded that alternative splicing events of ADD3 and cassette exon inclusion may potentially play a role in cancer progression. It is also noteworthy that cassette exon in ADD3 may have effects on its normal functions. The generation of the amino acids insert was predicted to form a small coiled coil motif upstream of the MARCKS-related region [179, 180]. Interestingly, both spliced isoforms of ADD3 were found in fusion with nucleoprotein 98 gene (NUP98) in patients with leukemia, and this translocation would also result in the formation of a chimeric protein involved in leukemogenesis [181, 182]. These studies suggested that the cancer-specific splicing transcript of adducin may play a role in tumorigenesis and can serve as a novel cancer biomarker.

## 6. Conclusion

Adducin is a major constituent of the membrane skeleton. Abnormal phosphorylation and genetic variants of adducin may disrupt membrane skeleton that in turn influence a variety of physiological processes and manifest in a variety of diseases. GWAS have revealed SNPs in adducin to be associated with particular clinical phenotypes although the effect size remains weak and further experimental studies are needed to elucidate the precise pathogenetic mechanisms involved. It appears that adducin genetic variants might contribute to aberrant protein phosphorylation and subsequent molecular alterations. Future studies that integrate SNPs information with related adducin phosphorylation would help to delineate its role in various diseases mentioned in this review. Moreover, the role of adducin in cancers remains unclear and controversial. More work should be done especially in an* in vivo* setting since cell-based studies may not fully reflect the properties of adducin in a disease-relevant environment. The differential splicing pattern of adducin in cancer should also be further investigated. This review provides a summary of adducin for its structure, regulation, and functions to pathogenic properties that can inform future basic and translational researches in this intriguing and exciting field of study.

## Figures and Tables

**Table 1:** Phosphorylation and calmodulin binding sites and the functional consequences.

	Phosphorylation sites	Functional consequences	References
Protein kinase A	*α*-Ser-726 *β*-Ser-713 *α*-Ser-408 *α*-Ser-436 *α*-Ser-481	Inhibit of actin-binding activity Inhibit spectrin-recruiting activity Inhibit calmodulin-binding activity Localize cell-cell contact in epithelial cells	[36, 53, 57]
	*α*-Ser-481	Stabilize endothelial adhesion junctions	[59]

Protein kinase C	*α*-Ser-726 *β*-Ser-713	Inhibit actin-capping activity Inhibit spectrin-recruiting activity Inhibit calmodulin-binding activity	[29, 37, 53]
	*β*-Ser-713 *β*-Ser-726	Nucleus translocation: support the structure of heterochromatin and centrioles during mitosis	[64]
	*α*-Ser-726	Promote membrane protrusions and cell motility	[58]
Protein kinase C and calpain	*α*-Ser-726 *γ*-Ser-656–668	Platelet activation and maintaining shape of resting platelet	[38, 56]

Rho-kinase	*α*-Thr-445 *α*-Thr-480	Enhance actin filament binding activity Enhance membrane ruffling Enhance cell motility	[54, 55]

Ca^2+^/calmodulin	*α*-718–734 *β*-425–444 *β*-705–721	Inhibit actin-capping activity Inhibit spectrin-recruiting activity Affect the rate of phosphorylation	[28, 33, 73]

**Table 2:** Phenotype characteristics associated with *Add*-null mice in different biological systems.

Gene knockout/allele symbol (synonym)	*Add1/Add1 tm1Llp*	*Add2/Add2 tm1Llp (β-adducin-)*	*Add3/Add3 tm1.2Llp (γ-add*Δ*ex*Δ*neo)*
Human disease model	Hydrocephalus	Hereditary spherocytosis type 1	Cerebral palsy, spastic quadriplegic, 3
	Essential hypertension		

Phenotypes	**Mortality/aging **	**Immune system**	**No abnormal phenotype detected**
	Prenatal lethality, incomplete penetrance	Increased spleen iron level	
	**Growth/size/body**	Enlarged spleen	-
	Decreased body size	**Liver/biliary system**	
	Decreased body weight	Increased liver iron level	
	**Hematopoietic system**	**Hematopoietic system**	
	Normal hematopoietic system phenotype	Anemia	
	Hemolytic anemia	Abnormal erythrocyte morphology	
	Abnormal erythrocyte morphology	Reticulocytosis	
	Thrombocytosis	Abnormal erythrocyte physiology	
	Decreased mean platelet volume	**Nervous system**	
	Reticulocytosis	Impaired synaptic plasticity	
	**Nervous system**	Abnormal CNS synaptic transmission	
	Nonobstructive hydrocephaly	Enhanced paired-pulse facilitation	
	**Homeostasis/metabolism**	**Renal/urinary system**	
	Normal homeostasis/metabolism phenotype	Increased kidney iron level	

References	[155]	[39]	[156]
